# Photoluminescence changes of C_70_ nano/submicro-crystals induced by high pressure and high temperature

**DOI:** 10.1038/srep38470

**Published:** 2016-12-06

**Authors:** Dedi Liu, Bingbing Liu, Bertil Sundqvist, Dapeng Dong, Zhenghua Li, Dongping Liu

**Affiliations:** 1 School of Physics and Materials Engineering, Dalian Minzu University, Dalian 116600, China; 2 State Key Laboratory of Superhard Materials, Jilin University, Changchun 130012, PR China; 3 Department of Physics, Umeå University, S-90187 Umeå, Sweden

## Abstract

Hollow C_70_ nano/submicro-crystals with a fcc lattice structure were treated under various high pressure and high temperature conditions. The energy band structure was visibly changed by the high pressure and high temperature treatment, and the luminescence of the treated C_70_ nano/submicro-crystals were tuned from the visible to the near infrared range. *In-situ* high pressure experiments at room temperature indicate that pressure plays a key role in the tuning of the band gap and PL properties in C_70_ nanocrystals, and temperature plays an important role in the formation of stable intermolecular bonds and thus to define the final red-shift of the PL peaks. The polymeric phases of C_70_ nanocrystals treated at high pressure and high temperature were identified from their Raman spectra, which showed a change from monomers to a dimer-rich phase and finally to a phase containing larger, disordered C_70_ oligomers.

Fullerene materials (C_60_, C_70_, etc.) constitute a new phase of carbon with distinctly different molecular, structural and electronic properties^1^. When the carbon cages are linked to each other forming polymeric structures, many novel physical and chemical properties appear, including optical, mechanical and electronic properties^2^,^3^,^4^. Fabricating polymeric fullerene materials is thus a productive and important way to obtain new functional materials. The high pressure and high temperature (HPHT) method has proved to be an efficient way to synthesize various kinds of polymeric fullerene phases. Earlier research on C_60_ crystals have shown the existence of one-dimensional orthorhombic (O) and two-dimensional tetragonal (T) and rhombohedral (R) polymer structures, consisting of C_60_ molecules joined by covalent 2 + 2 cycloaddition bonds^4^,^5^.

Another important member in the fullerene family, C_70_, can also be polymerized under HPHT conditions^5^,^6^,^7^. However, due to its special ellipsoidal molecular structure with only ten reactive double bonds, the polymerization of C_70_ under high pressure is not as easy as for C_60_. In one of the few existing studies, Soldatov *et al*. reported that a one-dimensional polymeric structure with a hexagonal lattice can be formed in monomeric hcp C_70_ crystals under relative low pressure^6^. The fcc structure is the most common crystal structure of C_70_, but it has long been assumed that C_70_ crystals with this lattice cannot form any long-range ordered polymeric structures because of the incompatible symmetries of the lattice and the molecular structure. Very recently, however, Marques *et al*.^8^,^9^ have shown that this barrier can be overcome by the application of sufficiently high pressures and temperatures. More work is certainly needed to answer the interesting and important basic scientific question whether larger oligomers than dimers can be formed in fcc structured C_70_ at reasonably low pressures.

Recently, the synthesis of fullerene nanocrystals with special physical and chemical properties has become a focus topic which is expected to lead to important applications in functional materials and devices. Our previous studies found many novel optical properties in polymerized C_60_ nanocrystals formed by the HPHT method^10^,^11^. In an earlier *in-situ* high pressure investigation on C_70_ nanocrystals we found that when the size of the C_70_ crystals were in the nano/submicrometer range many novel properties were observed, including higher phase transition pressures and a higher bulk modulus^12^, but no irreversible intermolecular bonding was found up to 43 GPa at room temperature. Intermolecular bonds are known to form in bulk C_70_ under HPHT conditions and the presence of such bonds might be expected to further modify the effects observed due to the nanosize effect and the molecular anisotropy. However, to the best of our knowledge no research on the polymerization of C_70_ nanocrystals under HPHT conditions has been reported that might help us understand the possible effects of intermolecular bonding on the properties of nano/submicrometer size C_70_ crystals. From a practical point of view, how the photoluminescence (PL) properties of C_70_ nanocrystals can be tuned by HPHT treatment is also an interesting question.

In this work, C_70_ nano/submicro-crystals with the fcc structure were fabricated by a solution method followed by heat treatment in vacuum^12^. Pristine C_70_ nanocrystals were treated under hydrostatic pressure conditions using a piston-cylinder device under different temperature and pressure conditions. The PL peaks of HPHT treated samples were tuned from the visible to the near infrared range. *In-situ* high pressure studies at room temperature elucidated the mechanism for the change in the band gap and luminescence of HPHT treated C_70_ nanocrystals. Raman spectroscopy was employed to map the evolution of intermolecular bonding in the C_70_ nanocrystals with the increase of temperature and pressure.

## Experiment section

Hollow C_70_ nano/submicro-crystals with the fcc structure were produced by a solution method described earlier^12^. X-ray diffraction measurements (XRD, Rigaku D/max-RA, CuKα1 radiation λ = 1.5418 Å) was used to characterise the lattice structure of the C_70_ nanocrystals.

The C_70_ nanocrystals produced were treated under different hydrostatic pressure conditions using a piston-cylinder device. In order to keep the initial morphologies of the C_70_ nanocrystals, we chose to use silicone oil (Dow Corning DC200) as pressure transmitting medium. The samples were heated to the final temperature at 0.5 GPa before final pressurization to avoid solidification of the oil. Also, for C_60_ we know that this procedure leads to a better phase purity of the polymeric phases. Three experiments were carried out under the conditions 2.0 GPa and 700 K for two, five and eight hours, respectively, to investigate possible phase transformations. The treated samples were then washed with pentane several times to remove the silicone oil after the treatment.

Several additional experiments were carried out to study the evolution of C_70_ under HPHT conditions and to investigate the mechanism for the change in PL of C_70_. First, C_70_ nanocrystals were treated at lower pressure and temperature, about 1.5 GPa and 573 K, and, second, *in-situ* high pressure PL studies of C_70_ nanocrystals were performed with a Diamond Anvil Cell (DAC) at room temperature.

Raman spectroscopy was used to investigate the lattice structure and the evolution of intermolecular bonding, and PL spectra were used to analyze the optical properties of all the HPHT treated samples. Both Raman and PL spectra were measured with a Renishaw inVia Raman spectrometer at room temperature, using the excitation line wavelengths of 514 nm. For comparison, the 830 nm laser was used in the Raman spectra acquisition of pristine samples, to identify the photopolymerization effect by the visible laser. A Renishaw 1000 Raman spectrometer with 830 nm laser was employed to investigate inter-cage vibrational (stretch) modes of polymerized fullerene. Scanning electron microscopy (SEM, SSX-550) was used to characterize the morphology of the C_70_ nanocrystals. The samples obtained were also characterized by transmission electron microscopy (TEM, JEM-2010, Japan).

## Results and Discussion

**Figure 1:**
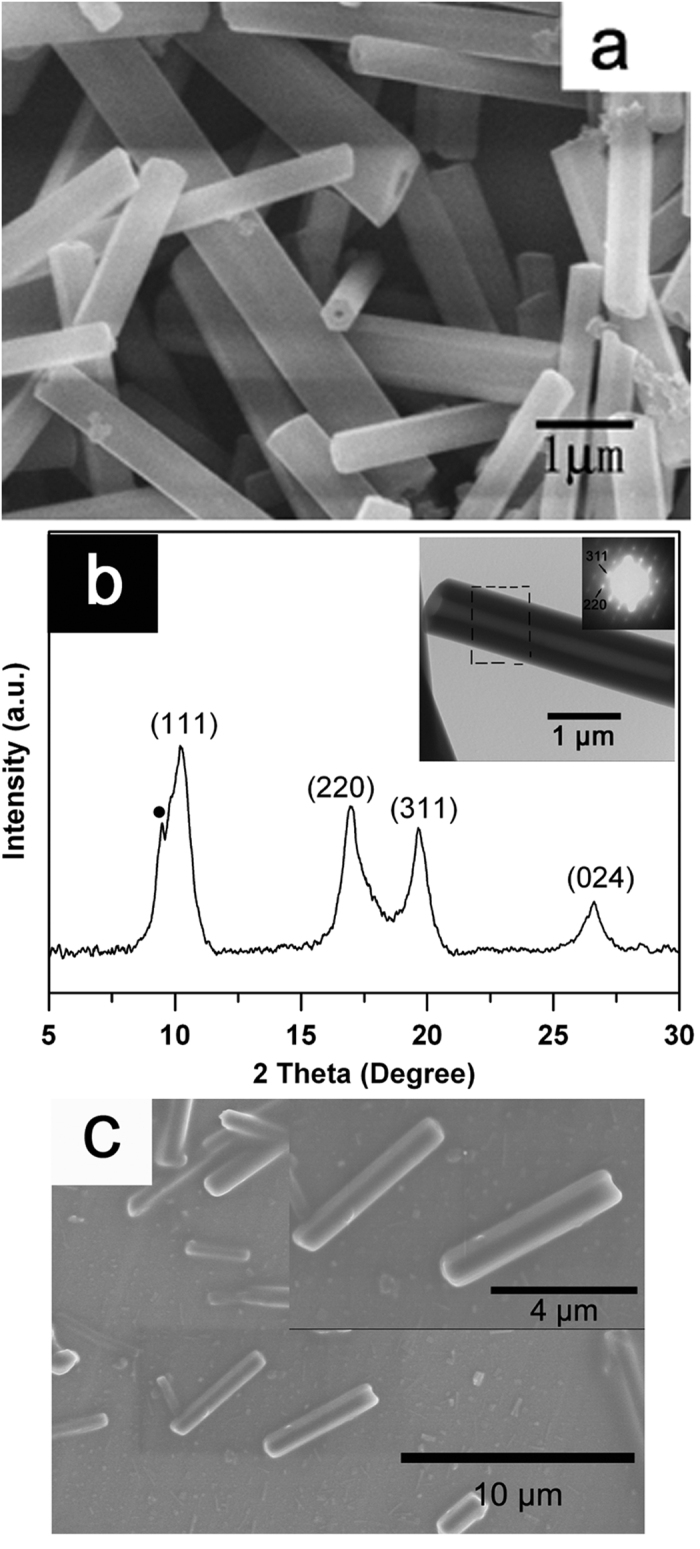
SEM image (**a**) and XRD pattern (**b**) of as-grown C_70_ nanocrystals, and SEM image of C_70_ nanocrystals after treatment under 2.0 GPa, 700 K (**c**). The insert in figure b shows the TEM image and SAED pattern of a single pristine C_70_ nanotube.

Figure 1a shows a SEM image of as-grown samples. The figure shows that the samples consist of nanocrystals with diameters in the range of 500–800 nm and lengths of about 4–10 μm. From the figure we can clearly observe that the cross-section of the obtained tubes have a hexagonal shape with round, empty channels along the tube axis. The TEM image shown in the insert of Fig. 1b clearly shows the empty channel of a single C_70_ nanocrystal, which further confirms the tubular structure of our sample.

To identify the crystal structure of the C_70_ nanocrystals, an XRD experiment was carried out on the pristine samples, and the XRD pattern obtained is shown in Fig. 1b. The diffraction peaks were indexed to be the (111), (220), (311) and (024) diffraction peaks of the fcc structure, with the lattice cell parameter a = 1.49 nm. This structure is similar to that of bulk crystals^13^,^14^. However, as the fcc structure of C_70_ nanocrystals was obtained after desolvation, the XRD curve is slightly different from that of bulk C_70_ crystals^14^. A strong shoulder before the (111) peak near 10 degrees probably arises from the presence of stacking faults, and a similar feature was also observed in previous reports on C_70_ bulk crystals. To confirm the structural results, a selected area electron diffraction (SAED) pattern from a single C_70_ nanotube is shown in the insert of Fig 1b. This SAED result is in good agreement with that from XRD, which indicates that the pristine C_70_ nanotubes had the fcc structure.

To verify that the C_70_ nanocrystals survived the HPHT treatment we produced SEM images of the HPHT treated samples with the results shown in Fig. 1c. Although the resolution is not sufficient to show any details, it is obvious that the sample is still in the form of individual, unbroken nano-sized tubes or rods similar in shape and size to the pristine material. This indicates that quasihydrostatic pressure treatment can keep the shape of the C_70_ nanocrystals.

Due to the ellipsoidal molecular structure, which gives a molecular symmetry lower than for C_60_, the luminescence of C_70_ is stronger than that of C_60_. It is thus even more interesting to study the optical properties of C_70_ nanocrystals for potential applications in the optical field. PL studies were thus carried out for pristine C_70_ nanocrystals and for the samples treated at 2 GPa, 700 K.

**Figure 2:**
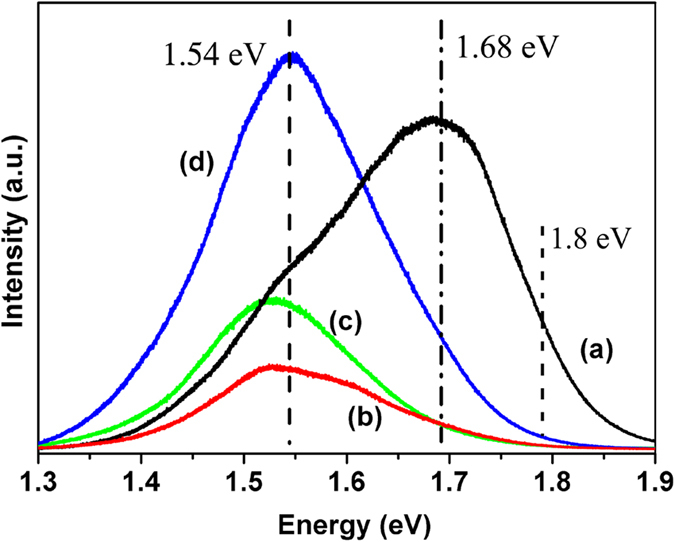
PL spectra of pristine C_70_ nanocrystals (**a**), and C_70_ nanocrystals treated at 2.0 GPa, 700 K for two, (**b**) five (**c**) and eight (**d**) hours, respectively.

Figure 2, curve a, shows the PL spectrum of pristine C_70_ nanocrystals. The PL energies typically range from 1.3 eV to 1.9 eV, with three dominant bands centered at 1.8 eV, 1.68 eV and 1.54 eV. The peak at 1.68 eV has the highest intensity. These PL bands and their energies are similar to previously reported results on C_70_ bulk samples and have been well discussed in both theory and experiments^15^,^16^,^17^,^18^. The peak at 1.8 eV is assigned to the radiative recombination of Frenkel-like excitons trapped at monomolecular defects near the sample surface, related to the purely electronic decay of the triplet T_1_ to the ground state S_0_ of the Frenkel-type polaron exciton^15^,^16^. The band centered at 1.68 eV originates from the recombination of excitons localized at defects consisting of adjacent C_70_ molecules^15^. However, the shoulder at 1.54 eV has not been well discussed or explained.

To investigate how the PL properties of C_70_ nanocrystals could be tuned by HPHT treatment, the samples treated at 2.0 GPa, 700 K for two, five and eight hours were characterized by PL spectroscopy. Their PL spectra are shown in Fig. 2, curves b,c and d, respectively. Obviously, a change appears in the PL spectra compared to that of pristine samples. In the PL spectra of the samples treated at 2.0 GPa, 700 K for different times, the maximum of the PL intensity moves to 1.54 eV and the peaks at 1.8 and 1.68 eV in the PL spectrum of pristine C_70_ nanocrystals could not be observed. This result means that the main luminescence of our C_70_ nanocrystals is tuned from the visible to the near infrared range with the HPHT method. This is helpful for applications in the biological and medicinal field because of the small auto-fluorescence from cells and biological tissue in this range^19^,^20^.

These phenomena indicated that the energy band structures of the C_70_ nanocrystals were changed after the HPHT treatment. The disappearance of the strongest peak at 1.68 eV is probably due to the formation of intermolecular bonds, which change the interaction of adjacent C_70_ molecules. The band gap of C_70_ crystals is known to decrease with increasing pressure^21^,^22^,^23^,^24^ and the band gap of C_60_ polymers decreases with an increasing number of intermolecular bonds per molecule^5^. These results suggest that HPHT treatment is an effective way to change the band gap and luminescence properties of C_70_ nanocrystals. However, these measurements did not allow us to determine whether dimers or long chain polymers were formed under these conditions and the detailed evolution of the shift in the PL was still unclear. We return to these questions below.

**Figure 3:**
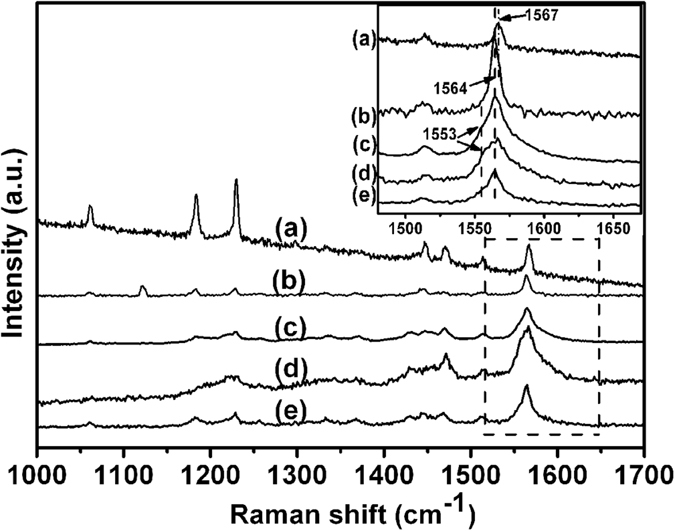
Top two curves: Raman spectra of pristine C_70_ nanocrystals using 830 nm (**a**) and 514 nm (**b**) lasers as excitation line, respectively. Lower three curves: Raman spectra, obtained using 514 nm laser excitation, from C_70_ nanocrystals treated at 2.0 GPa, 700 K for two, (**c**) five (**d**) and eight (**e**) hours, respectively.

Raman spectroscopy is a powerful tool to characterize polymeric phases of fullerenes, since the C_60_/C_70_ intermolecular bond configuration strongly influences the Raman spectrum. Due to the reduced symmetry of the C_70_ molecule compared to the C_60_ molecule, the number of Raman allowed vibrational modes of this molecule is much larger. For pure C_70_ at room temperature, 53 Raman active modes are predicted (12A_1_′ +22E_2_′ +19E_1_′) from the D_5h_ point group according to group theory. In our experiment, Raman spectroscopy was used to characterize the structural phases of C_70_ nanocrystals after HTHP treatment. The Raman spectra of as-grown C_70_ nanocrystals obtained using 830 nm and 514 nm lasers as excitation lines are shown in Fig. 3, curve a and b, respectively. In both curves, more than seven peaks were found in the range 1000 cm^−1^ to 1700 cm^−1^, in excellent agreement with earlier results on pristine C_70_ bulk material^15^,^16^. This further verified that the nanocrystals consist of pure C_70_. However, a slight difference was observed for the characteristic peak at 1567 cm^−1^ obtained using the 830 nm laser. When the 514 nm laser was used, a slight red shift to1564 cm^−1^ was observed for this peak and only a shoulder could be observed at 1567 cm^−1^. This could probably be due to the slight photo-polymerization induced by the 514 nm laser.

To study the structural phases of the C_70_ nanocrystals treated at 2.0 GPa, 700 K for different times, Raman spectra of the relevant samples were again examined. The Raman spectra of C_70_ nanocrystals treated for two hours are shown in Fig. 3, curve c. In previous literature^6^,^7^, a splitting of the characteristic peak at 1567 cm^−1^ was observed by Soldatov’s group with the 1064 cm^−1^ 6, and red-shift were observed in most of other investigations on polymerized phases of bulk C_70_^7^. By direct comparison with the Raman spectrum of pristine C_70_ nanocrystals in the insert, we can see that an obvious broadening of this characteristic peak occurs after HPHT treatment, and the peak centers for all the HPHT treated C_70_ nanocrystals are at 1564 cm^−1^. Notably, a weak peak at 1553 cm^−1^ appears beside this strongest peak as a shoulder. These phenomena indicated that intermolecular bonds were formed in the HPHT treated samples, but whether larger oligomers were formed in our C_70_ nanocrystals is still unclear.

To investigate whether longer oligomers could occur in our C_70_ nanocrystals, the samples were treated for longer time to reach a more complete polymerization state. New pristine samples were treated for five and eight hours, respectively. The Raman spectra of these two samples are shown in Fig. 3, curves d and e. A comparison of these two curves with curve c shows no obvious differences. This is very similar to what was observed for the PL results. The results indicate that it is impossible to produce long oligomers (one-dimensional chain-like polymerized phases) at these pressures when starting from fcc C_70_ crystals. This is most probably due to the initial molecular orientations in our nanocrystals, which is known to make formation of the zigzag type polymer chains impossible for symmetry reasons.

**Figure 4:**
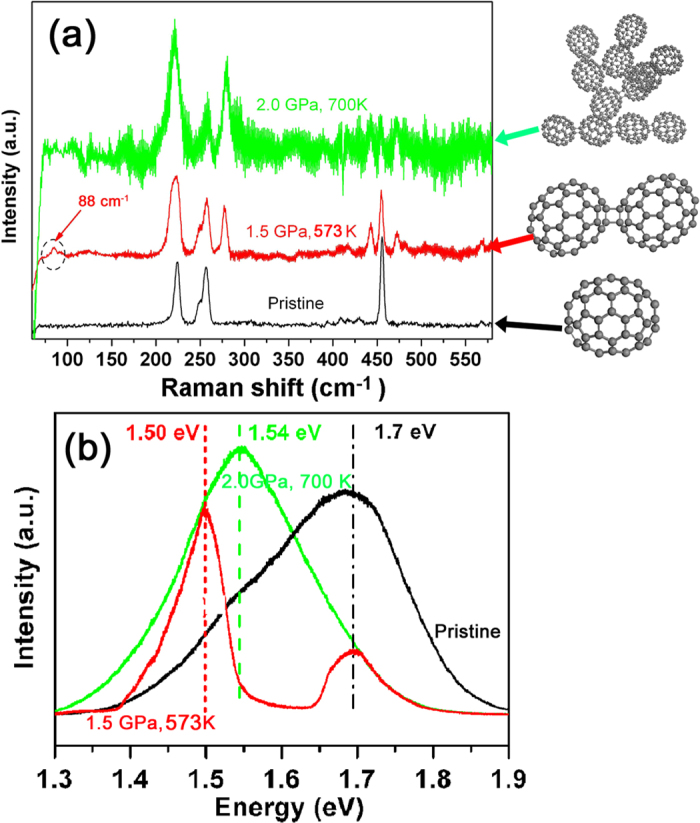
Raman (**a**) and PL (**b**) spectra of pristine C_70_ nanocrystals. and of C_70_ nanocrystals after treatment under the conditions of 1.5 GPa, 573 K and 2 GPa, 700 K. respectively. In (**a**) we also show the structures of C_70_ monomers and dimers, and a sketch showing a possible structure for a disordered oligomer.

In the above discussions, the formation of intermolecular bonds of C_70_ molecules is mainly determined from the shift or split of the intramolecular Raman modes in high-frequency area, but this method provides no information about the lattice. However, for C_70_ dimers and chains the inter-cage vibrational (stretch) modes of polymerized fullerene, which indicate that the heavy fullerene cages move as rigid units connected by the intermolecular bonds, should show up as low-frequency Raman peaks^6^,^7^,^25^. To further confirm the formation of C_70_ oligomers, low-frequency Raman spectra of HPHT treated C_70_ nanocrystals were detected with an excitation wavelength of 830 nm. In Fig. 4a, we compare Raman spectra from pristine C_70_ nanocrystals and from C_70_ nanocrystals treated at 1.5 GPa, 500 K and 2 GPa, 690 K. Compared to the pristine samples, several changes in the Raman features are clearly seen: (i) Many peaks broaden due to disorder. (ii) The strong, sharp “monomer” peaks near 260 and 455 cm^−1^ diminish at 1.5 GPa and nearly disappear at 2 GPa. (iii) A new peak, characteristic for dimers and polymers^6^,^7^, appears near 275 cm^−1^. It is strong after treatment at 1.5 GPa and even stronger after treatment at 2 GPa. (iv) Finally, and most important, the dimer stretching mode at 88 cm^−1^ is clearly seen after treatment at 1.5 GPa.

All these features verify that dimeric or polymeric structures have formed after treatment under both these HTHP conditions, and that the polymerization degree increases with the increase of pressure and temperature.

In previous works, the longitudinal lattice modes of linear C_70_ chains was reported near 105 cm^−1^ 6, and the C_70_ dimer stretching mode was found at 89 cm^−1^ 7. These modes cannot exist for monomers and they are thus clear fingerprints for the dimer or polymer states. In our investigation a sharp new line was found near 88 cm^−1^ for the samples after treatment at 1.5 GPa. It is in the right place and with the right relative intensity for a stretching mode. Together with the high intensity of the new mode near 275 cm^−1^ this indicates that the samples treated at 1.5 GPa contained a relatively high concentration of C_70_ dimers. In a recent report, room-temperature dimerization was found at the much higher pressure of 7.5 GPa^26^. The fact that we find a high concentration of dimers already at 1.5 GPa shows that it is much easier to obtain C_70_ dimers under high temperature conditions.

However, no stretching mode was observed for the samples treated at 2 GPa. It is reasonable to assume that when the pressure and temperature increase, more and more fullerene molecules form intermolecular bonds, and this is verified by the strong growth of the “dimer/polymer” line near 275 cm^−1^ and the almost complete elimination of the lines at 260 and 455 cm^−1^, known to be characteristic for the C_70_ monomer^6^,^7^. We believe that the reason why we do not see any stretching mode for the samples treated at 2 GPa is that the polymerization reaction has proceeded too far, such that dimers have grown or coalesced into larger, disordered C_70_ oligomers. In a recent report, it was concluded that this type of structures did not give any stretching modes for C_60_ materials^25^. We show in Fig. 4a some drawings of the suggested molecular structures to help the reader to visualize these. Clearly, in a structure of the type shown at the top, no strong intermolecular vibrations could be excited.

In previous literature, many HPHT investigations on fullerene peapods (fullerene molecules inserted into carbon nanotubes) were reported^27^,^28^,^29^. Both one-dimensional polymerization and dimerization of C_60_ have been reported inside peapods, like in bulk materials. However, C_70_ cannot be induced to form even dimers in C_70_-peapod samples, because an applied pressure forces the C_70_ molecules inside peapods into positions quite unfavorable for dimerization or polymerization. These studies verify that the formation of C_70_ polymers and dimers requires exacting geometrical considerations. In this study, C_70_ dimers were obtained in nanocrystals at the conditions of 1.5 GPa and 573 K, but when the pressure and temperature increased only disordered oligomers could be obtained. Again, this indicates that it is not possible to form ordered C_70_ oligomers from the fcc phase even in C_70_ nanocrystals.

To further explore the relationship between the PL properties and polymerization, we measured the PL spectrum for the samples after the intermediate treatment at 1.5 GPa, 573 K (shown as Fig. 4b). When the samples were treated under these conditions the PL band split into two peaks at 1.70 eV to 1.50 eV, respectively. According to the Raman spectra, the C_70_ nanocrystals changed from a monomer structure to a dimer-rich phase. As shown above, when the samples were treated at 2.0 GPa 573 K, the PL peak changed to single centered peak at 1.54 eV. The Raman indicated that these samples probably contained a large volume fraction of disordered C_70_ oligomers. All these results indicate that the PL properties are closely related with the polymerization state, which also suggests that the PL peak of C_70_ nanocrystals can be controlled by tuning their polymeric phases.

**Figure 5:**
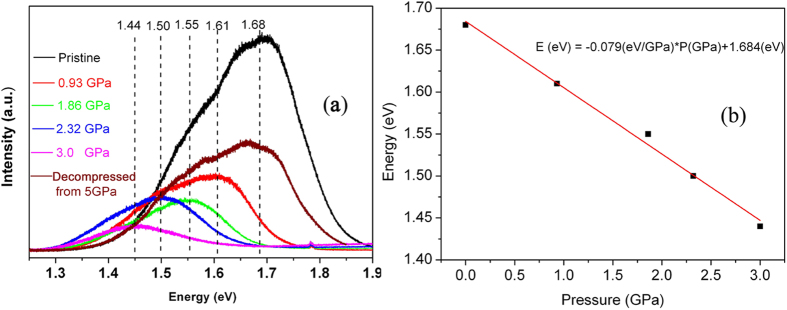
*In-situ* PL spectra of C_70_ nanocrystals up to 3 GPa (**a**) and pressure dependence of the peak center positions of the PL spectra for C_70_ nanocrystals (**b**).

To identify the evolution of and the key factors for the change in the PL properties of C_70_ nanocrystals, *in situ* PL spectra of C_70_ nanocrystals under pressure were also recorded, using a DAC (diamond anvil cell). Figure 5a shows the PL spectra at the relatively low pressures of 0, 0.93, 1.86, 2.32 and 3.0 GPa. With increasing pressure, the PL bands move in the direction of larger wavelengths and the intensities of the PL bands become weaker. The redshift of the PL spectra indicate that the band gap of C_70_ is reduced with increasing pressure, which suggests that the interaction between the C_70_ molecules is enhanced by the effect of pressure.

To determine the factors influencing the formation of intermolecular bonds in C_70_ nanocrystals, the pressure dependence of the PL centers below 3 GPa is shown in Fig. 5b. As shown in this figure, the energy E at which the maximum occurs for the C_70_ PL peaks varies linearly with increasing pressure, and fitting a straight line to the data we find the pressure dependence dE/dP = −0.079 eV/GPa. Because the PL is closely related to the band gap, we can compare this value with the recent results of Thirunavukkuarasu *et al*.^26^. They measured the pressure dependence of the band gap from the position of the absorption edge E_g_ and found a value of dEg/dP = −0.077 eV/GPa. According to our data, when the pressure is 2 GPa, the PL maximum should appear at about 1.53 eV. This value is in close proximity to that of our HPHT treated samples (1.54 eV). This result indicated that pressure plays a key role in the tuning of the band gap and the PL properties, while temperature has no large effect. When the pressure was higher than 5 GPa, the PL peak becomes too weak to be observed. To investigate the luminescence properties of C_70_ nanocrystals after this pressure cycle, we also recorded the PL spectra from samples released from 5 GPa, after removal from the diamond anvil. As shown in Fig. 5a, the PL peak position for these samples is almost the same as that for pristine C_70_ nanocrystals. These results indicate that the shift of the PL peaks with pressure is reversible at room temperature. However, as discussed above the PL spectra did not return to their initial state after the HPHT treatment. Thus, we verify that the temperature plays an important role in the process by enabling the formation of a sufficient number of intermolecular bonds to lock the C_70_ molecules into a state containing a very large volume fraction of stable C_70_ disordered oligomers. In this state, which is metastable at ambient pressure, the intermolecular interaction is strong enough to produce a shift in the PL spectrum equivalent to an applied pressure of 2 GPa. In a much simplified picture we could describe this as “locking in” an internal pressure of 2 GPa in the crystal by the formation of random intermolecular “polymer” bonds, resulting in a stable red shift of the PL spectra.

## Conclusion

In summary, we have tuned the PL properties of C_70_ nanocrystals using the HPHT method. After treatment under 2.0 GPa and 700 K for 2 hours, 5 hours and 8 hours, respectively, the center of the main PL band for all the samples shifted from 1.68 eV to 1.54 eV, a significantly lower energy than that for pristine C_70_ nanocrystals. In contrast, *in-situ* high pressure PL studies show that pressure alone could only cause a reversible change of the band gap in C_70_ nanocrystals. High temperature plays a key role in the formation of a stable polymeric phase and an irreversible change of the band gap. Systematic Raman spectroscopy studies indicated that C_70_ nanocrystals with an initial fcc structure first transform to a dimer-rich phase, and then to a disordered oligomer-rich phase. Long one-dimensional polymer chains could not be obtained from nanocrystals with the fcc structure. This study thus clarifies the evolution of the formation of C_70_ disordered oligomers and the mechanism for the luminescence change in HTHP treated C_70_ nanocrystals.

## Additional Information

**How to cite this article**: Liu, D. *et al*. Photoluminescence changes of C_70_ nano/submicro-crystals induced by high pressure and high temperature. *Sci. Rep.* **6**, 38470; doi: 10.1038/srep38470 (2016).

**Publisher's note:** Springer Nature remains neutral with regard to jurisdictional claims in published maps and institutional affiliations.
